# Inter-Mode Crosstalk Estimation between Cores for *LP_mn_* Modes in Weakly Coupled Few-Mode Multicore Fiber with Perturbations

**DOI:** 10.3390/s24185969

**Published:** 2024-09-14

**Authors:** Shuangmeng Liu, Lian Xiang

**Affiliations:** 1 School of Electronic and Information Engineering, Soochow University, Suzhou 215006, China; lxiang@suda.edu.cn; 2 Faculty of Business Information, Shanghai Business School, Shanghai 201400, China

**Keywords:** inter-mode crosstalk, few-mode multicore fiber, mode coupling coefficient

## Abstract

A novel inter-mode crosstalk (IMXT) model of ${LP}_{mn}$ mode for weakly coupled few-mode multicore fiber is proposed based on the coupled mode theory (CMT) with bending and twisting perturbations. A universal expression of the mode coupling coefficient (MCC) between ${LP}_{mn}$ modes is derived. By employing this MCC, the universal semi-analytical model (USAM) of inter-core crosstalk (ICXT) can be applied to calculate the IMXT. Simulation results show that our model is generally consistent with previous theories when stochastic perturbations are absent. Moreover, our model can work effectively when stochastic perturbations are present, where former theories are not able to work properly. It has been theoretically found that the MCC has an intimate relationship with core pitch. Our model, based on the CMT, can provide physical characteristics in detail, which has not been reported clearly by former theories. In addition, our model is applicable to phase-matching and non-phase-matching regions of both real homogeneous and heterogeneous few-mode multicore fibers (FM-MCFs) with a wider range of applications.

## 1. Introduction

Multicore fibers (MCFs) based on spatial division multiplexing (SDM) technology can greatly alleviate the capacity limitations of single-mode fiber (SMF) [1,2,3,4]. Transmission media for SDM are mostly multicore fiber and few-mode fiber (FMF) [5]. Nowadays, few-mode multicore fibers (FM-MCFs) [6] significantly enhance transmission capacity as a hybrid method of MCF and FMF. In past studies, a data rate of 5.1 Tb/s per carrier has been reached using hole-assisted FM-MCF over a 1 km single fiber with seven few-mode cores [7,8]. However, inter-core crosstalk (ICXT) is one of the most important influencing factors in coupled MCF, which significantly degrades transmission performance [9,10,11,12]. Therefore, quite a few researchers have focused on analyzing the characteristics of ICXT evolution caused by variations in fiber structures and external factors [13,14,15].

In recent years, various theoretical and experimental studies have been reported in the literature to accurately characterize and model ICXT. In [16], a discrete changes model (DCM) for the longitudinal evolution of ICXT in homogeneous weakly coupled MCFs with bending and twisting perturbations was proposed based on the coupled mode theory (CMT). As a typical model for ICXT estimation, a DCM works well in the phase-matching region, but it is not applicable to the non-phase-matching region and cannot be used for heterogeneous MCFs. Although continuously enhanced DCMs have also been reported subsequently, they still cannot operate in the non-phase-matched region [13,14,15,16,17]. In [18], the coupling coefficient between cores has been derived analytically to evaluate ICXT with single mode. Based on the analytical coupling coefficient, a universal semi-analytical model (USAM) of ICXT for real coupled MCFs has been put forward, which can be utilized both in phase-matching and phase-mismatching regions [19]. So far, a highly mature theoretical foundation has been developed in the study of ICXT estimation for multicore single-mode fibers. Typically, the CMT and the coupled power theory (CPT) are widely employed for investigating the estimation of ICXT [20,21].

Similarly, inter-mode crosstalk (IMXT) plays the same role in FM-MCF [22]. Research on IMXT for FM-MCF is still in its initial stages. In [23], the effect of inter-core polarization mode dispersion (PMD) on IMXT was studied, and a closed expression for the correlation length of the birefringence vector was given. In [24], IMXT was derived from stochastic differential equations (SDEs) based on the CPT, which considered both deterministic and stochastic inter-core coupling. That work found that when the deterministic coupling strength was sufficiently strong, it could suppress the influence of the PMD on IMXT for ${LP}_{mn}\;$ modes. However, the IMXT method in [24] did not consider the impact of bending and twisting perturbations on crosstalk. In addition, the impact of random perturbations on crosstalk was not considered in [24], which re-characterizes IMXT in practical FM-MCF transmission [25,26].

In this paper, we derive a universal expression of the mode coupling coefficient (MCC) for ${LP}_{mn}$ modes between cores based on the CMT and Maxwell equations to estimate the IMXT in FM-MCF. The theoretical derivation of the MCC allowed us to precisely define the relationship between the deterministic coupling effect and the stochastic coupling effect mentioned in [24], and to obtain accurate IMXT values, whereas previous models have used mode coupling coefficient ($K_{mn}$) approximation, which leads to large errors. In addition, we analyzed the optimal line segment length for IMXT estimation in theory. In the next simulation, we found that the value of IMXT is greatly affected by stochastic perturbations, which cannot be estimated by previous theories. Therefore, our theory can provide a quite reliable model for the IMXT simulation of weakly coupled FM-MCF with random perturbations. Additionally, our model is influenced by physical parameters like core pitch and optical wavelength, which has not been addressed in previous theories. Generally, this paper is structured as follows. In Section 2, we discuss the derivation of the MCC and IMXT in detail. In Section 3, firstly, we present numerical simulations conducted to verify our theory. Next, we investigate the influence of IMXT on stochastic perturbations and physical characteristics. In Section 4, we present our conclusion.

## 2. Analysis and Methods

In this section, firstly, the MCC for the modes is derived from Maxwell equations. Next, an expression for IMXT estimation is obtained by employing the CMT. In ${LP}_{mn}$ mode, *m* means that the pattern satisfies a Bessel function of order *m*, and *n* means that there are *n* solutions of that order. Our model can be applied to estimate the IMXT not only for ${LP}_{o1}$, but also for higher order modes.

### 2.1. Mode Coupling Coefficient of ${LP}_{mn}$ Mode

The definition of mode coupling coefficient for ${LP}_{mn}$ modes between cores can be written as [27]:

(1) $$k_{mn}=\frac{\omega \varepsilon_{0}\iint \left(n_{1}^{2}-n_{2}^{2}\right)E_{m}^{*}\cdot E_{n}^{*}dxdy}{\iint e_{z}\cdot \left(E_{m}^{*}\times H_{m}\right)+E_{m}\times H_{m}^{*})dxdy}$$

where ω is the angular frequency, $\varepsilon_{0}$ is the free-space permittivity, and $n_{1}$ and $n_{2}$ represent indexes of core and cladding, respectively. ${\;e}_{z}$ represents a unit vector for x-polarization. $E_{m}$ and $H_{m}$ are electric and magnetic fields in the core domain, respectively, and $E_{n}$ is the electric field in the cladding domain. The symbol * indicates the conjugate transform. According to [27], the denominator of (1) is related to the optical power.

(2) $$\iint e_{Z}\cdot \left({E_{m}^{*}\times H}_{m}\right)+E_{m}\times H_{m}^{*})dxdy=4P$$

### 2.2. Electric Field of ${LP}_{mn}$ Mode Based on Maxwell Equations

As the fiber is a cylindrical structure, in column polar coordinates the electric field of the LP mode can be written as [27]:

(3) $$E=e_{r}E_{r}+e_{\varphi }E_{\varphi }+e_{z}E_{z}$$

where $E_{x}$ denotes the electric field component in the x-direction and $e_{x}$ denotes the unit vector in the x-direction. The transverse electric field component $E_{r}$ and $E_{\varphi }$ is difficult to obtain, but the longitudinal component can be solved by the chi-squared Helmholtz equation in [27] as follows:

(4-1) $$E_{z1}=AJ_{m}\left(\frac{U}{a}r\right)\cos\left(m\varphi \right),0\leq r\leq a\;\;\;\;\;\;\;\;\;\;\;\;\;\;$$

(4-2) $$E_{Z2}=A\frac{J_{m}\left(U\right)}{K_{m}\left(W\right)}K_{m}\left(\frac{W}{a}r\right)\cos\left(m\varphi \right),r\geq a$$

(5-1) $$E_{r1}=-j\frac{a^{2}}{U^{2}}\left[\frac{\beta UA}{a}J_{m}^{\prime }\left(\frac{U}{a}r\right)+\frac{\omega {µ}_{0}mB}{r}J_{m}\left(\frac{U}{a}r\right)\right]\cos\left(m\varphi \right),0\leq r\leq a$$

(5-2) $$E_{r2}=j\frac{a^{2}}{W^{2}}\frac{J_{n}(U)}{K_{n}(W)}\left[\frac{\beta WA}{a}K_{m}^{\prime }\left(\frac{W}{a}r\right)+\frac{\omega {µ}_{0}mB}{r}K_{m}\left(\frac{W}{a}r\right)\right]\cos\left(m\varphi \right),r\geq a$$

(5-3) $$E_{\varphi 1}=-j\frac{a^{2}}{U^{2}}\left[-\frac{\beta mA}{r}J_{m}\left(\frac{U}{a}r\right)-\frac{\omega {µ}_{0}UB}{a}J_{m}^{\prime }\left(\frac{U}{a}r\right)\right]\sin\left(m\varphi \right),0\leq r\leq a$$

(5-4) $$E_{\varphi 2}=j\frac{a^{2}}{W^{2}}\frac{J_{m}(U)}{K_{m}(W)}\left[-\frac{\beta mA}{r}K_{m}\left(\frac{W}{a}r\right)-\frac{\omega {µ}_{0}WB}{a}K_{m}^{\prime }\left(\frac{W}{a}r\right)\right]\sin\left(m\varphi \right),r\geq a$$

where $J_{m}(x)$ means the m order Bessel function of the first kind and $K_{m}(x)$ means the m order modified Bessel function of the second kind. *U* and *W* are the normalized transverse phase and attenuation parameters, respectively. $U=a\sqrt{k^{2}n_{1}^{2}-\beta^{2}}$ and $W=a\sqrt{\beta^{2}-k^{2}n_{2}^{2}\;}$, where β is the propagation constant, a is the core radius, and k=2π/λ is the wave number, where λ is wavelength. A=jUC/aβ, where *C* is a system constant mentioned in [27].$B=-jUC/\omega u_{0}a$, where $J_{m}^{\prime }\left(x\right)$ is the first derivative of $J_{m}(x)$ and $K_{m}^{\prime }(x)$ is the first derivative of $K_{m}(x)$.

The electric field of ${LP}_{mn}$ behaves as a superposition of three dimensions:

(6) $$\begin{array}{c} \;\;\;I_{m}=E_{z1}^{*}E_{z2}+E_{r1}^{*}E_{r2}+E_{\varphi 1}^{*}E_{\varphi 2}=\frac{J_{m}\left(U\right)}{K_{m}\left(W\right)}J_{m}\left(\frac{U}{a}r\right)K_{m}\left(\frac{W}{a}r\right)\\ \;\;\left(A^{2}{cos}^{2}\left(m\varphi \right)-\frac{m^{2}a^{4}\omega^{2}{µ}_{0}^{2}B^{2}}{U^{2}W^{2}r^{2}}{cos}^{2}\left(m\varphi \right)-\frac{m^{2}a^{4}\beta^{2}A^{2}}{U^{2}W^{2}r^{2}}{sin}^{2}\left(m\varphi \right)\right)\\ -\frac{J_{m}\left(U\right)}{K_{m}\left(W\right)}J_{m}^{\prime }\left(\frac{U}{a}r\right)K_{m}^{\prime }\left(\frac{W}{a}r\right)(\frac{a^{2}\beta^{2}A^{2}}{UW}{cos}^{2}\left(m\varphi \right)+\frac{a^{2}\omega^{2}{µ}_{0}^{2}B^{2}}{UW}{sin}^{2}\left(m\varphi \right))\\ -\frac{J_{m}\left(U\right)}{K_{m}\left(W\right)}J_{m}^{\prime }\left(\frac{U}{a}r\right)K_{m}\left(\frac{W}{a}r\right)\frac{a^{3}\beta Am\omega {µ}_{0}B}{{UW}^{2}r}\\ -\frac{J_{m}\left(U\right)}{K_{m}\left(W\right)}J_{m}\left(\frac{U}{a}r\right)K_{m}^{\prime }\left(\frac{W}{a}r\right)\frac{a^{3}\beta Am\omega {µ}_{0}B}{U^{2}Wr} \end{array}$$

**Figure 1:**
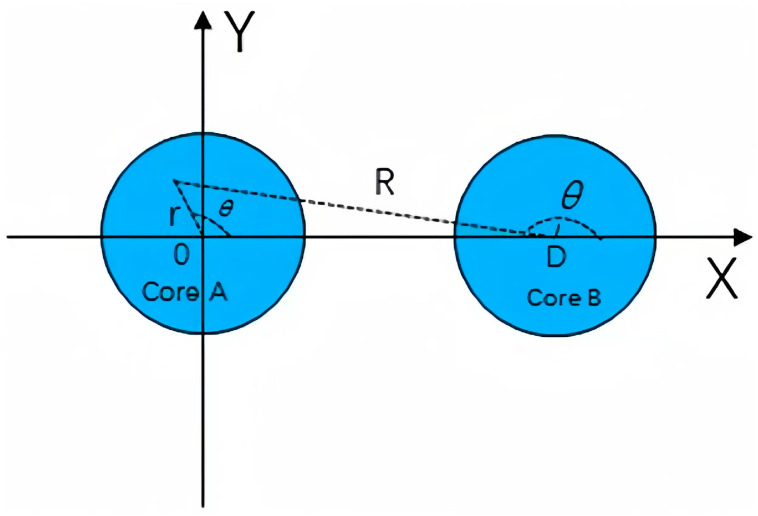
Schematic diagram of few-mode two-core fiber.

Here, we set the value of *m* to obtain the electric field superposition of the corresponding ${LP}_{mn}$ mode. Next, bringing (6) to the numerator of (1), we obtain

(7) $$S_{m}=\iint {(n}_{1}^{2}-n_{0}^{2})E_{m}^{*}\cdot E_{n}^{*}dxdy=\iint (n_{1}^{2}-n_{0}^{2})I_{m}rdxd\varphi$$

where (*R,*θ) is a coordinate system originating at the center of core two, which represents the domain of *r > a*, as shown in Figure 1. The core pitch is denoted by *D*. When *D >> r* holds, radius *R* can be approximated as $R=\sqrt{D^{2}+r^{2}-2Drcos(\theta )}≌D-rcos(\theta )$.

The final expression for the ${LP}_{mn}$ mode coupling coefficients is obtained by bringing $S_{m}$ into (1):

(8) $$k_{mn}=\frac{\omega \varepsilon_{0}S_{m}}{4P}$$

### 2.3. Inter-Mode Crosstalk Based on CMT

Based on the CMT, coupled-mode equations in coupled FM-MCFs can be expressed as [27]:

(9) $$\frac{dE}{dz}=j\beta E+jkE$$

where E and β are the electric field matrix and the propagation constant matrix, respectively. k is a mode coupling coefficient matrix made up with $k_{mn}$. A universal semi-analytical model (USM) has been proposed to solve the coupled mode equation in [19]. Therefore, we can generally evaluate the IMXT as:

(10) $$IMXT=\sum_{i=1}^{N}IMXT_{i}=\sum_{i=1}^{N}\frac{\frac{k_{mn,i}^{2}}{g_{mn,i}^{2}}{sin}^{2}\left(g_{mn,i}d\right)}{{cos}^{2}\left(g_{mn,i}d\right)+\frac{\Delta \beta_{mn,i}^{2}}{{4g}_{mn,i}^{2}}{sin}^{2}\left(g_{mn,i}d\right)}\;\;\;\;\;\;\;=\sum_{i=1}^{N}{\left[\frac{k_{mn,i}}{g_{mn,i}}\sin\left(g_{mn,i}d\right)\right]}^{2}$$

where $g_{mn,i}=\sqrt{k_{mn,i}^{2}+(\frac{\Delta \beta_{mn,i}}{2})}$ means modified mode coefficient, $k_{mn,i}$ is computed by (1) and $\Delta \beta_{mn,i}$ means the equivalent phase mismatching, which is defined in [19] as:

(11) $$\Delta \beta_{mn,i}\left(d\right)=\beta_{m,i}\left(d\right)-\beta_{n,i}(d)$$

where $\beta_{m,i}\left(d\right)$ and $\beta_{n,i}\left(d\right)$ are equivalent propagation constants of core m and n, respectively, defined as:

(12) $$\beta_{i}(d)\approx \beta_{c}\beta_{p}[R_{b}+rcos\theta \left(d\right)]/R_{b}$$

where $\beta_{c}=\left(2\pi /\lambda \right)n_{eff}^{\left(int\right)}$ is the unperturbed propagation constant of the fiber core, and $n_{eff}^{\left(int\right)}$ is the intrinsic effective refractive index of the fundamental mode. *β_p_* represents the longitudinal fluctuations of propagation constants caused by inherent and external fluctuations. $R_{b}$ is the bending radius. $\theta \left(d\right)=\gamma d+\varphi$, where γ and φ represent the twist rate of the core and the offset of the twist, respectively.

**Figure 2:**
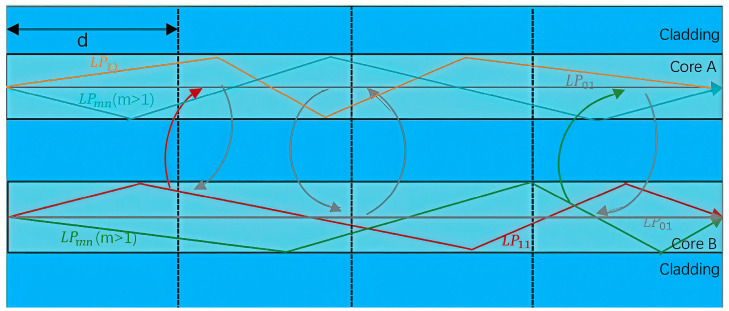
Schematic of mode coupling in few-mode two-core fiber.

Based on the principle of USM, fully considering the characteristics of the stochastic perturbation, the fiber length is divided into N segments with each segment length d, as shown in Figure 2.

## 3. Results and Discussion

**Figure 3:**
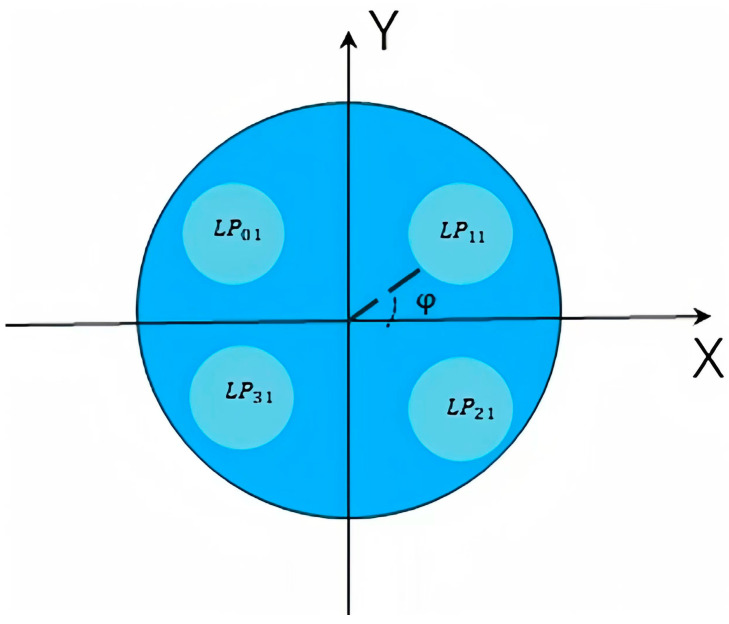
Schematic diagram of few-mode four-core fiber.

In this section, the two-core fiber in [24] and four-core fiber are discussed in detail. Schematic diagrams of two-core fiber and four-core fiber are shown in Figure 1 and Figure 3, respectively. Firstly, for two-core fiber, numerical simulations were carried out without stochastic perturbations to determine the optimal segment length and verify the accuracy of our model by comparison with the Monte Carlo Simulation in [23] and the analytical expression in [24]. In addition, the impact of physical characteristics, such as core pitch and optical wavelength, are discussed. Next, IMXT characteristics of the four-core fiber are studied. In a four-core fiber, it is assumed that one mode exists in each core, the ${LP}_{01}$ mode exists in core 1, and ${LP}_{1n\;},{LP}_{2n},$ and ${LP}_{3n}$ modes exist in core 2, core 2, and core 3, respectively. In this paper, we refer to the evaluation method of [24] and use symmetric mode–asymmetric mode inter-mode coupling to approximate the inter-mode coupling as a generic *LP* mode. The fundamental mode of core 1 is used as the symmetric mode, and other low-order modes of core 2, core 3, and core 4 are used as asymmetric modes.

### 3.1. Fiber Parameter

For a few-mode two-core fiber, the core radius is 2.5 um, the cladding index is 1.45, and the wavelength is 1550 um, which are the same as those in [24]. For a few-mode four-core fiber, the IMXT between core 1 and core 2, core 1 and core 3, and core 1 and core 4 are discussed. To satisfy the normalization requirement, detailed parameters for the four-core fiber are shown in Table 1.

### 3.2. IMXT Analysis of Two-Core Few-Mode Fiber

**Figure 4:**
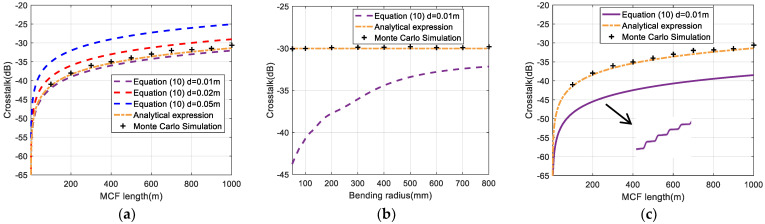
IMXT as a function of (**a**) FM−MCF length without stochastic perturbations, (**b**) bending radius, and (**c**) FM−MCF length with bending and twisting perturbation.

The two-core fiber was simulated first. The evaluation of IMXT is shown in Figure 4. Figure 4a shows the simulation results of IMXT as a function of the FM-MCF length for different segment lengths in the absence of stochastic perturbations. When segment length d=0.01 m, the IMXT obtained by our theory matched well with the Monte Carlo Simulation in [23] and the analytical expression in [24], as shown by the purple dotted line, orange dotted dashed line, and black crosses in Figure 4a. When segment length d=0.02 m and d=0.05 m, the IMXT obtained from our theory was strikingly different from those of the former models. It is worth noting that the precision of the IMXT model is strongly dependent on segment length. It was shown that the segment length d=0.01 m can be seen as an optimal segment length. So, the simulation results of our theory presented in the rest of our work were obtained with segment length d=0.01 m.

Furthermore, when the effects of bending and twisting perturbations in (12) are taken into account, it shows the IMXT as a function of bending radius for our model compared with former models when FM-MCF length is set to 1000 m, as shown in Figure 4b. The IMXT is significantly suppressed by bending perturbation, especially at a small bending radius. However, the former models cannot work normally with bending perturbation. A similar conclusion can be obtained with twisting and bending perturbations, as shown in Figure 4c, where $R_{b}=0.2\;m$,γ=2π rad/m. Moreover, the suppression on IMXT enhances with an FM-MCF length increase. Results in Figure 4b,c illustrate that both the Monte Carlo Model [23] and the analytical expression [24] are not sensitive to bending and twisting perturbations. However, in Figure 4c, we can see that our model reduces the IMXT value by 8 dB at 1000 m MCF length, which is because stochastic perturbations increase the effective refractive index difference between cores, which makes the IMXT computed by our theories lower than those of former theories. As the crosstalk increases significantly at the phase-matching point, some fluctuations in the purple curve estimated by our model can be found with changes over the distribution of the phase-matching point [17]. Therefore, our model can well reflect the effects of perturbations on IMXT.

**Figure 5:**
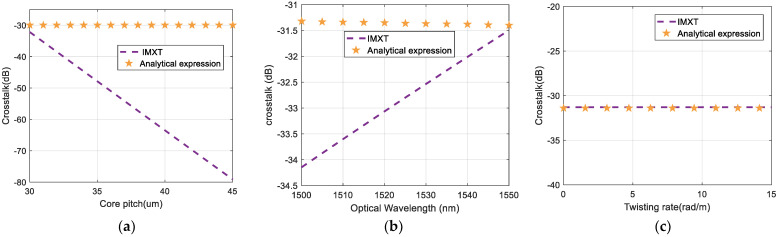
IMXT as a function of (**a**) core pitch, (**b**) optical wavelength, and (**c**) twisting rate for IMXT and analytical expression.

In addition, former theories have not discussed the relationship between IMXT with physical characteristics. Figure 5 shows simulation results of IMXT as a function of core pitches, optical wavelengths, and twisting rates for our IMXT model and the analytical expression in [24]. Orange pentagrams represent the analytical expression based on CPT and purple dotted lines represent our IMXT model, which takes into account the impact of $k_{mn}$. These results indicate that the fiber parameters of our model have an effect on the estimation of IMXT under stochastic perturbations, which is similar to that of ICXT at MCF. However, the fiber parameters in the analytical expression do not have much effect on IMXT. This is because the analytical expression takes an approximation for $k_{mn}$, whereas our model for $k_{mn}$ performs a detailed derivation.

### 3.3. IMXT Analysis of Four-Core Few-Mode Fiber

**Figure 6:**
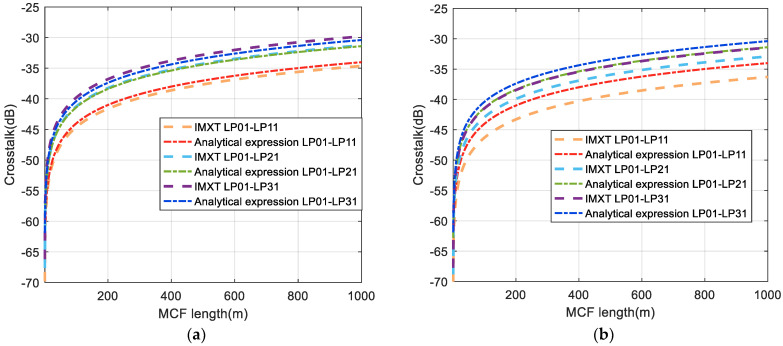
Estimation of crosstalk from the ${LP}_{01}$ to the ${LP}_{11}({LP}_{21},{LP}_{31}$) as a function of FM−MCF length (**a**) without stochastic perturbations and (**b**) with stochastic perturbation.

Thereafter, the four-core FMF was simulated. See Appendix A for specific derivation. We took the ${LP}_{01}$ mode as the longitudinal electric field distribution and other modes as the transverse electric field distribution to evaluate and analyze the crosstalk of the modes. Simulation results are shown in Figure 6. The dotted and dotted dashed lines in the figure represent the IMXT between the fundamental and other higher-order modes in our model and the analytical expression [24], respectively. In Figure 6a, it can be seen that crosstalk between the different modes obtained by our model and the analytical expression could be well matched under no stochastic perturbations. However, in the presence of stochastic perturbation, it can be clearly seen that crosstalk values between different modes obtained by our model became significantly smaller, whereas crosstalk obtained by the analytical expression had no obvious effect, as shown in Figure 6b. The trend of this simulation is consistent with the one obtained above using two-core fiber.

**Figure 7:**
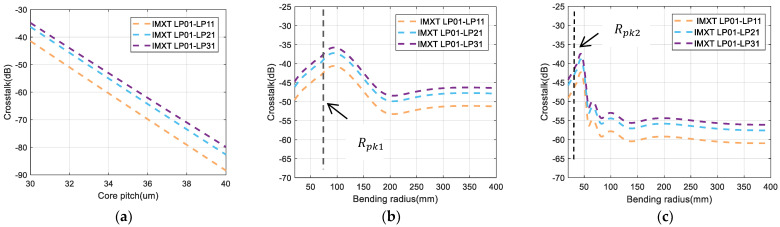
Estimation of crosstalk from ${LP}_{01}$ to ${LP}_{11\;}({LP}_{21},{LP}_{31}$*)* as a function of (**a**) core pitch, (**b**) bending radius in homogeneous FM−MCF, and (**c**) bending radius in heterogeneous FM−MCF.

In the above study of the physical parameters of two-core fiber, we know that in the analytical expression the fiber parameters did not have a great influence on the IMXT. So, we directly studied our model subject to the core pitch and bending radius on the IMXT as shown in Figure 7. Figure 7a shows the IMXT as a function of the core pitch for our model, and it can be seen that IMXT continued to decrease as the inter-core distance increased. When the distance between the cores increased, MCCs decreased and the coupling effect between the modes decreased, resulting in a decrease in IMXT. Figure 7b,c show the IMXT as a function of the bending radius for our model in homogeneous and heterogeneous FM-MCFs. For values $\Delta n_{eff,mn}^{\left(int\right)}=0.020\%$ and $\Delta n_{eff,mn}^{\left(int\right)}=0.042\%$, the threshold bending radius $R_{PK1}=96\;mm$ and $R_{PK2}=43\;mm$, respectively. Simulation results of the actual homogeneous few-mode four-core fiber are shown in Figure 7a. In the phase-matching region, IMXT is proportional to the bending radius. However, in the non-phase-matching region, IMXT is inversely proportional to the bending radius. The value of the crosstalk decreased with the bending radius and tended to a stable value gradually. Next, Figure 7b illustrates the heterogeneous few-mode four-core fiber with a larger $\Delta n_{eff,mn}^{\left(int\right)}$. These results show that the variation trend is basically the same as that shown in Figure 7a. The trend of these results is similar to the theoretical analysis of ICXT in MCF [19,28,29].

## 4. Conclusions

In this paper, we study the model of stochastic IMXT for ${LP}_{mn}$ mode in weakly coupled FM-MCFs with random perturbations based on the CMT and Maxwell equations. In the absence of random perturbations, our model can well match the Monte Carlo Simulation [23] and the analytical expression [24] at optimal segment length d=0.01 m, which verifies the accuracy of our model. In addition, our model calculates $K_{mn}$ accurately, which can effectively minimize experimental error and make up for the shortcomings of previous studies that take into account only the approximate value of $K_{mn}$. In the presence of bending perturbations, our model more accurately estimates the effect of bending radius on the FM-MCF. The IMXT is about 8 dB lower than those of previous models, which is due to the fact that previous models directly ignored bending perturbations, which is not realistic. Next, we investigated the effects of physical properties, such as core pitch and optical wavelength, on IMXT. Results show that the IMXT of the fiber can be mitigated by rationally configuring the physical structure of the fiber, which has not been discussed in previous models. Notably, study of the bending radius revealed that the model is applicable to both phase-matching and non-phase-matching regions of both homogeneous and heterogeneous FM-MCFs. Overall, we propose a systematic theory for IMXT estimation, which is more widely applicable and more accurately calculated in practical FM-MCF transmission with stochastic perturbations.

## Figures and Tables

**Table 1:** Parameters of the four-core fiber.

Parameters	Symbol	Value
Core pitch	D	30 μm
Core radius	a	8 μm
Core index	$n_{1}$	1.4530
Cladding index	$n_{0}$	1.444
Wavelength	λ	1550 nm
Bending radius	$R_{b}$	400 mm
Twisting rate	γ	2π rad/m

## Data Availability

Datasets presented in this article are not readily available because these data are part of an ongoing study.

## Appendix A

In a four-core FMF, the fundamental mode is used as the longitudinal electric field distribution such that *m* = 0 in (4):

(A1) $$E_{z1}=AJ_{0}\left(\frac{U}{a}r\right),0\leq r\leq a\;\;\;\;\;$$

(A2) $$E_{Z2}=A\frac{J_{0}\left(U\right)}{K_{0}\left(W\right)}K_{0}\left(\frac{W}{a}r\right),r\geq a$$

Other modes are used as transverse electric field distributions, which are obtained by making *m =* 1 in (5) when calculating the crosstalk between the ${LP}_{01}$ and ${LP}_{11}$ modes:

(A3-1) $$E_{r1}=-j\frac{a^{2}}{U^{2}}\left[\frac{\beta UA}{a}J_{1}^{\prime }\left(\frac{U}{a}r\right)+\frac{\omega {µ}_{0}B}{r}J_{1}\left(\frac{U}{a}r\right)\right]\cos\left(\varphi \right),0\leq r\leq a$$

(A3-2)
$$\;E_{r2}=j\frac{a^{2}}{W^{2}}\frac{J_{1}(U)}{K_{1}(W)}\left[\frac{\beta WA}{a}K_{1}^{\prime }\left(\frac{W}{a}r\right)+\frac{\omega {µ}_{0}B}{r}K_{1}\left(\frac{W}{a}r\right)\right]\cos\left(\varphi \right),r\geq a$$

(A3-3)
$$\;\;\;\;\;\;\;\;E_{\varphi 1}=-j\frac{a^{2}}{U^{2}}\left[-\frac{\beta A}{r}J_{1}\left(\frac{U}{a}r\right)-\frac{\omega {µ}_{0}UB}{a}J_{1}^{\prime }\left(\frac{U}{a}r\right)\right]\sin\left(\varphi \right),0\leq r\leq a$$

(A3-4)
$$E_{\varphi 2}=j\frac{a^{2}}{w^{2}}\frac{J_{1}(U)}{K_{1}(W)}\left[-\frac{\beta A}{r}K_{1}\left(\frac{W}{a}r\right)-\frac{\omega {µ}_{0}WB}{a}K_{1}^{\prime }\left(\frac{W}{a}r\right)\right]\sin\left(\varphi \right),r\geq a$$

The electric field is then superimposed:

(A4) $$I_{m}=E_{z1}^{*}E_{z2}+E_{r1}^{*}E_{r2}+E_{\varphi 1}^{*}E_{\varphi 2}=A^{2}\frac{J_{0}\left(U\right)}{K_{0}\left(W\right)}J_{0}\left(\frac{U}{a}r\right)K_{0}\left(\frac{W}{a}r\right)\;-\frac{J_{1}\left(U\right)}{K_{1}\left(W\right)}J_{1}^{\prime }\left(\frac{U}{a}r\right)K_{1}^{\prime }\left(\frac{W}{a}r\right)(\frac{a^{2}\beta^{2}A^{2}}{UW}{cos}^{2}\left(\varphi \right)+\frac{a^{2}\omega^{2}{µ}_{0}^{2}B^{2}}{UW}{sin}^{2}\left(\varphi \right))\;-\frac{J_{1}\left(U\right)}{K_{1}\left(W\right)}J_{1}^{\prime }\left(\frac{U}{a}r\right)K_{1}\left(\frac{W}{a}r\right)\frac{a^{3}\beta A\omega {µ}_{0}B}{{UW}^{2}r}\;-\frac{J_{1}\left(U\right)}{K_{1}\left(W\right)}J_{1}\left(\frac{U}{a}r\right)K_{1}^{\prime }\left(\frac{W}{a}r\right)\frac{a^{3}\beta A\omega {µ}_{0}B}{U^{2}Wr}$$

Substituting $I_{m}$ into (7) to continue the theoretical derivation, we obtain the crosstalk between the modes of ${LP}_{01}\_{LP}_{11}$. Following this derivation, the crosstalk between the modes of ${LP}_{01}\_{LP}_{21}$ and ${LP}_{01}\_{LP}_{31}$ can be obtained.
